# Unusual congenital malformation of the larynx

**DOI:** 10.5935/1808-8694.20130092

**Published:** 2015-10-08

**Authors:** Renata Santos Bittencourt Silva, Marco Antonio dos Anjos Corvo, Larissa Kallas Curiati, Renata Christofe Garrafa, Claudia Alessandra Eckley

**Affiliations:** a Third-year Resident Physician, Department of Otorhinolaryngology, Santa Casa de São Paulo.; b MSc. in Otorhinolaryngology, Medical Sciences School of the Santa Casa de São Paulo, Doctoral Student, Medical Sciences School of the Santa Casa de São Paulo. (Assistant Professor, Department of Otorhinolaryngology, Santa Casa de São Paulo).; c Fifh-year Medical Student, Medical Sciences School of the Santa Casa de São Paulo.; d Sixth-year Medical Student, Medical Sciences School of the Santa Casa de São Paulo.; e PhD in Otorhinolaryngology, Medical Sciences School of the Santa Casa de São Paulo (Assistant Professor, Department of Otorhinolaryngology, Santa Casa de São Paulo). Medical Sciences School of the Santa Casa de São Paulo.

**Keywords:** congenital abnormalities, laryngeal diseases, larynx, respiratory tract abnormalities

## INTRODUCTION

Congenital anomalies rank among the top causes of stridor in neonates and infants. Laryngomalacia stands out as a frequent causing agent, along with less prevalent occurrences of laryngeal membrane, laryngeal atresia and subglottic stenosis^1^, ^2^, ^3^. Laryngeal membranes are thin translucent formations consequent to failed laryngeal lumen recanalization during embryogenesis^1^, ^4^. Most laryngeal membranes are located in the anterior larynx, and account for 5% of laryngeal malformations^5^.

Anterior laryngeal membrane has been described as a persistent wider horizontal membrane with a posterior central orifice located between the vocal folds^4^. Clinical findings include biphasic stridor with dyspnea and weak cry, and severity correlates with lesion extension^4^. When the obstruction produced by the membrane is complete, patients are diagnosed with laryngeal atresia, a life-threatening condition^4^. Nonetheless, most congenital airway anomalies are not severe when not accompanied by acute comorbidities leading to respiratory involvement^6^.

## CASE PRESENTATION

The patient was a full-term vaginal birth male child with history of heart malformations without hemodynamic repercussions. At the age of four months, the patient had upper respiratory tract infection (URTI) followed by bronchiolitis. He was admitted at another hospital for 15 days and underwent intensive care and orotracheal intubation for seven days. The medical team who saw the patient reported he had not been intubated previously.

**Figure 1:**
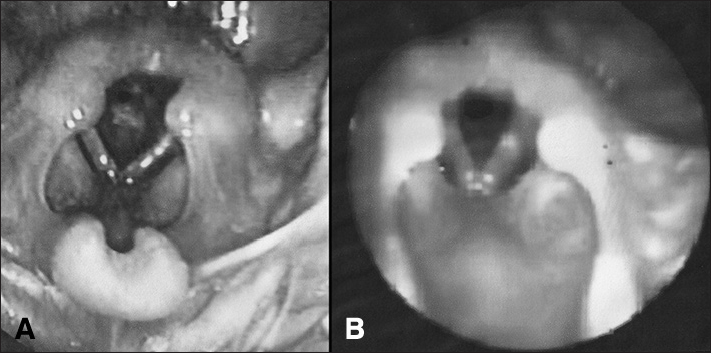
Laryngeal membrane in a child aged four months. A: Extensive laryngeal membrane producing tight evenly shaped round posterior glottic lumen; B: Endoscopic examination nine days after dilation procedure showing notable increase in glottic lumen despite remnants of congenital malformation.

Seven days after discharge, the patient was taken to our service with respiratory effort and biphasic laryngeal stridor. Examination revealed a heart rate of 110 bpm, a respiratory rate of 58 breaths per minute, subcostal retractions, biphasic stridor, and oxygen saturation of 91%. Chest x-rays showed hyperinflated lungs; CBC did not show infection; test results were negative for acute phase inflammation. The patient underwent laryngoscopy on the first day of admission. A thin but extensive subglottic membrane was visualized, producing a round central posterior glottic cleft measuring approximately 2.0 mm (Figure 1A). The patient was promptly submitted to endoscopic dilation using 12-16 catheters. Minimal bleeding occurred during the procedure, and the patient's respiratory pattern and stridor improved immediately. He was kept only on support therapy for an additional nine days of hospitalization. Fiberoptic endoscopy performed nine days after surgery revealed increased glottic lumen, despite the presence of remnants of laryngeal membrane (Figure 1B).

## DISCUSSION

Laryngeal malformations are more prevalent in males and manifest within the first months of life^2^, ^3^, ^6^. The laryngeal involvement seen in the case presented extended from the anterior to the posterior direction, had a round central-posterior lumen, and was accompanied by signs consistent with an uncommon and potentially severe case of laryngeal membrane. Despite the lack of information on the details of the patient's previous hospitalization, the short period he spent on orotracheal intubation, the purely glottic location of the lesion, and the membrane's structural characteristics were used to rule out other diagnostic possibilities such as subglottic stenosis and post-intubation injury. Synechiae in more posterior locations are common in post-intubation laryngeal injuries, given the position of the tube in the respiratory portion of the larynx. Their shape is notably uneven and signs of trauma are evident^2^, ^3^. Additionally, the frequent association of heart malformations and congenital laryngeal involvement reinforce the innate character of the lesion^3^.

The chosen endoscopic approaches were indispensable both in the diagnosis and treatment of the patient's condition. Endoscopic resections have been broadly used to produce extremely satisfactory outcomes^2^. Dilation may be useful when performed early in the development of the disease^2^. The use of cauterization, cryosurgery, and CO_2_ laser has been described with varying degrees of success^3^. Dilation catheters were used in this patient because the membrane was thin and no subglottic involvement was observed. Despite the remnants of laryngeal membrane, the patient improved significantly and was kept on long-term follow-up for possible future revision procedures.

## CLOSING REMARKS

Laryngeal membrane is an uncommon congenital disease characterized by biphasic stridor within the first months of life. Accurate identification of the lesion led to prompt choice of therapy, favorable prognosis, and ultimately patient survival free of respiratory complications.
